# Interaction of HP1 and Brg1/Brm with the Globular Domain of Histone H3 Is Required for HP1-Mediated Repression

**DOI:** 10.1371/journal.pgen.1000769

**Published:** 2009-12-11

**Authors:** Marc Lavigne, Ragnhild Eskeland, Saliha Azebi, Violaine Saint-André, Suk Min Jang, Eric Batsché, Hua-Ying Fan, Robert E. Kingston, Axel Imhof, Christian Muchardt

**Affiliations:** 1 Institut Pasteur, Département de Biologie du Développement, Unité de Recherche Associée URA2578 du Centre National de la Recherche Scientifique CNRS, Unité de Régulation Epigénétique, équipe AVENIR de l'Institut National de la Santé Et de la Recherche Médicale INSERM, Paris, France; 2 Munich Center for Integrated Protein Science CIPSM, Histone Modifications Group, Adolf-Butenandt Institute, Ludwig-Maximilians University of Munich, Munich, Germany; 3 Department of Molecular Biology, Massachusetts General Hospital, Boston, Massachusetts, United States of America; Max-Planck-Institute of Immunobiology, Germany

## Abstract

The heterochromatin-enriched HP1 proteins play a critical role in regulation of transcription. These proteins contain two related domains known as the chromo- and the chromoshadow-domain. The chromo-domain binds histone H3 tails methylated on lysine 9. However, *in vivo* and *in vitro* experiments have shown that the affinity of HP1 proteins to native methylated chromatin is relatively poor and that the opening of chromatin occurring during DNA replication facilitates their binding to nucleosomes. These observations prompted us to investigate whether HP1 proteins have additional histone binding activities, envisioning also affinity for regions potentially occluded by the nucleosome structure. We find that the chromoshadow-domain interacts with histone H3 in a region located partially inside the nucleosomal barrel at the entry/exit point of the nucleosome. Interestingly, this region is also contacted by the catalytic subunits of the human SWI/SNF complex. *In vitro*, efficient SWI/SNF remodeling requires this contact and is inhibited in the presence of HP1 proteins. The antagonism between SWI/SNF and HP1 proteins is also observed *in viv*o on a series of interferon-regulated genes. Finally, we show that SWI/SNF activity favors loading of HP1 proteins to chromatin both *in vivo* and *in vitro*. Altogether, our data suggest that HP1 chromoshadow-domains can benefit from the opening of nucleosomal structures to bind chromatin and that HP1 proteins use this property to detect and arrest unwanted chromatin remodeling.

## Author Summary

HP1 proteins are transcriptional regulators frequently associated with gene silencing, a phenomenon involving masking of promoter DNA by dense chromatin. Owing to their chromo-domain, these proteins can read and bind an epigenetic mark that on many non-expressed genes is present on histone H3 at the surface of the nucleosome (the fundamental packing unit of chromatin). However, the binding to this mark does not explain the repressing activity of HP1 proteins. Here, we show that these proteins can establish a second contact with histone H3, independently of the epigenetic mark. This second contact site is located inside the nucleosome, in a position likely to be inaccessible. Interestingly, this site is also contacted by a subunit of the SWI/SNF complex and this contact is required for the ATP-dependent chromatin remodeling catalyzed by SWI/SNF. We provide evidence suggesting that HP1 proteins use the SWI/SNF chromatin remodeling to gain access to the contact site inside the nucleosome and to prevent further remodeling by competing with SWI/SNF for binding at this position. These observations lead us to suggest that HP1 proteins function as gatekeepers on promoters, detecting and stopping unwanted exposure of internal nucleosomal sites.

## Introduction

HP1 proteins are important regulators of heterochromatin-mediated silencing and chromosome structure in diverse eukaryotes (for recent reviews, see [1],[2]). In mammalian cells, the HP1 family is composed of HP1α, HP1β, and HP1γ. So far, only HP1β has been inactivated in the mouse, resulting in defective development of neuromuscular junctions and cerebral cortex [3]. Within the nucleus, the three HP1 isoforms all concentrate in foci of dense pericentromeric heterochromatin but are also present in the rest of the nucleoplasm. Consistent with this very general distribution, the mammalian HP1 proteins are detected not only in dense heterochromatic regions but also on active euchromatic genes [4].

On these active genes, HP1 proteins seem to be present both during phases of silencing and transcriptional activity. For example, on the Survivin gene and on an integrated HIV1 LTR, HP1β is detected on the repressed promoter, while HP1γ is recruited after transcriptional activation [5],[6]. HP1γ is however not always associated with active transcription as it participates in the repression of the MMTV LTR and the Sox2 promoter [7],[8]. It is noteworthy also that on the HIV1 LTR and on the β-major gene, HP1 proteins are co-localized with the RNA polymerase II (RNAPII), indicating that they are not creating an environment incompatible with recruitment of this polymerase [5],[9]. Taken together, these observations suggest that, at least in euchromatin, HP1 proteins are not “chromatin condensers” *per se*, but more likely regulators of enzymatic activities involved in transcription initiation or elongation.

HP1 proteins contain two very similar domains known as the chromo-domain (CD) and the chromoshadow-domain (CSD) separated by a less structured hinge region. The CSD is required for dimerization and interaction with many molecular partners that share a PXVXL motif [10]. It is also necessary for the recruitment of HP1 proteins to sites of DNA damage [11]. Concurrently, the CD recognizes and binds histone H3 tails methylated on lysine 9 (K9), an epigenetic mark frequently associated with transcriptional repression [12],[13]. In addition, the hinge region of HP1 proteins harbors DNA- and RNA-binding activities and the targeting of these proteins to chromatin likely results from the integration of multiple contacts [14],[15].

In contrast to the strong binding to peptides mimicking histone H3 tails methylated on K9, HP1 proteins bind only weakly to reconstituted methylated nucleosomal arrays [16]. Consistent with this, binding of HP1 proteins to purified native chromatin *in vitro* seems relatively inefficient [15],[17]. This binding can be improved by auxiliary factors that may help the recognition of chromatin [16], but it has also been suggested that HP1 can benefit from chromatin opening. Indeed, a more stable incorporation of HP1 proteins occurs in S phase when DNA replication disrupts the histone octamers [17]. Earlier reports also describe the presence in the nucleus of two populations of HP1 proteins with either high or low mobility [18] and it has been proposed that the more stable interaction creates the HP1 population of low mobility [3]. Binding of HP1 proteins may also benefit from ATP-dependent chromatin remodeling as HP1β co-localize with the ACF1-ISWI remodeling complex [19]. In addition, HP1α, but not HP1β and HP1γ, interacts with Brg1 and Brm, the mutually exclusive catalytic subunit of the human SWI/SNF (hSWI/SNF) complex, and this interaction favors repression of a reporter construct by a transfected Gal4-HP1α fusion protein (Figure S1A, S1B, S1C, S1D and [20],[21]).

To gain better understanding of HP1 chromatin binding and transcriptional regulation, we have here examined whether these proteins could establish alternative interactions with the histones. This allowed us to identify a contact between the CSD and a region of histone H3 located at the border of the globular domain. This region is also contacted by the hSWI/SNF subunits Brg1 and Brm, and we show that HP1 proteins have a negative effect on hSWI/SNF-mediated chromatin remodeling. Finally, we provide evidence indicating that hSWI/SNF activity is involved in the recruitment of HP1 proteins to chromatin.

## Results

### The chromoshadow-domain interacts with the globular domain of histone H3

**Figure 1:**
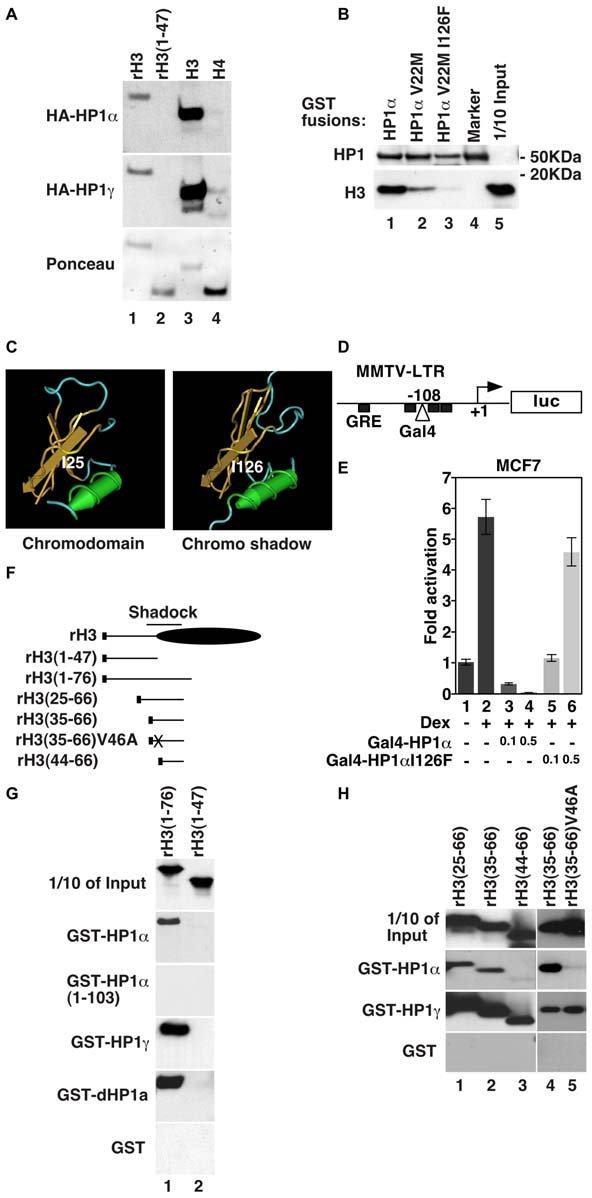
The CSD of HP1 proteins is a histone-binding domain. (A) Indicated histones either epitope-tagged recombinant (rH3) or purified bovine (H3 and H4) were resolved by SDS-PAGE, blotted to nitrocellulose membrane and probed with either HA-HP1α or HA-HP1γ. Bound HP1 proteins were detected with anti-HA antibodies and chemiluminescence. (B) Purified bovine core histones were incubated with indicated HP1α-derived GST-fusion proteins bound to agarose beads. After washing, retained proteins were eluted, resolved by 4–12% gradient SDS-PAGE, and detected by western blotting with anti-histone H3 antibodies. (C) Isoleucine 25 and 126 (I25 and I126) positioned on the structure of the chromo-domain (1Q3L) and the monomeric chromoshadow-domain (1DZ1) respectively, visualized with CN3D. (D) Schematic representation of the MMTV(Gal4)-Luc reporter construct. Black boxes symbolize glucocorticoid receptor (GR) binding sites (GRE). (E) MCF7 cells were transfected with 1mg of the Gal4-MMTV-Luc reporter construct in the absence or in the presence of dexamethasone (Dex – induces activation of the MMTV promoter by the glucocorticoid receptor) and the indicated amounts (in µg) of Gal4-HP1α or Gal4-HP1αI126F expression constructs. (F) Schematic representation of the B10/6xHIS-tagged recombinant histone H3 constructs expressed in *E. coli*. Black boxes represent the B10/6xHIS tag. (G,H) Indicated histone H3 mutants were incubated with indicated HP1-derived GST-fusion proteins and probed for interaction as in (B). Western blotting was performed with anti-B10 monoclonal antibodies.

We investigated whether HP1 proteins could bind histone H3 independently of the well-characterized association of the CD with methylated K9. To this end, we tested the binding of HP1α and HP1γ to either purified or recombinant B10-epitope-tagged histones immobilized on nitrocellulose membrane. As expected, the HP1 proteins bound strongly to purified histone H3 but not to histone H4 (Figure 1A, lanes 3 and 4). Interestingly, we also observed weaker but significant binding to full-length recombinant histone H3 produced in *E. coli* and therefore not methylated on K9 (Figure 1A, lane 1). This binding was not observed on the tail region alone (Figure 1A, lane 2). This is in accordance with earlier studies showing interaction of HP1 proteins with the globular domain of recombinant histone H3 [17],[22]. In GST pull down assays, we also observed weaker, but persisting histone H3 binding after mutation of the CD at position V22, abolishing interaction of HP1α with the methylated histone H3 tail (Figure 1B, lane 2). This again suggested the presence of additional contact points between HP1α and histone H3.

The structure of the CSD is very similar to that of the CD (Figure 1C), prompting us to probe for an interaction with the histones via this domain. To this end, we further mutated HP1α V22M at position I126 inside the CSD. This position is equivalent to I25 in the CD, an amino acid that, when mutated, prevents the domain from interacting with histone H3 [22]. This position was chosen because V22 has no equivalent in the CSD. The double mutant no longer interacted with H3, indicating that in both the CD and the CSD, the first β strand is involved in histone interaction (Figure 1B, lane 3). Mutation of the CSD at I126 also affected the repressing activity of HP1α. This was visualized by co-transfecting in MCF7 cells an MMTV/Gal4 reporter construct and expression plasmids for Gal4-HP1α fusions where HP1α was either WT or with an I126F mutation (Figure 1D and 1E).

We next mapped the region of histone H3 involved in the interaction with the HP1α CSD. A non-modified histone H3 truncation mutant spanning from aa 1 to 76 produced in *E.coli* was sufficient to interact with HP1α, while a shorter construct containing only the H3 tail region (aa 1–47) failed to do so. The interaction was disrupted by deletion of the CSD, confirming its implication in the contact with H3 (Figure 1F and 1G, 3 top panels). We noted also that all mutations affecting HP1 dimerization abolished the CSD-H3 interaction, while this interaction resisted incubation with a DNA intercalating agent (data not shown). This series of experiments also showed that HP1γ and *Drosophila* dHP1a had binding activities similar to that of HP1α (Figure 1G, bottom panels).

We finally identified aa 35 to 66 as the minimal region binding both HP1α and HP1γ (Figure 1H, lane 2 and 4). We termed this region the Shadock for “chromoShadow docking”. This region contains a PXXVXL motif resembling the PXVXL motif frequently found in proteins interacting with the CSD of HP1 proteins [10]. Mutation of the valine in this sequence (V46) abolished binding to HP1α but not HP1γ (Figure 1H, lane 5). Consistent with this, the Shadock could be further shortened to aa 44 to 66 without disrupting binding of HP1γ (Figure 1H, lane 3). These observations show that the two proteins have overlapping but not identical binding sites.

### Brg1, Brm, and HP1 contact overlapping regions in the globular domain of histone H3

**Figure 2:**
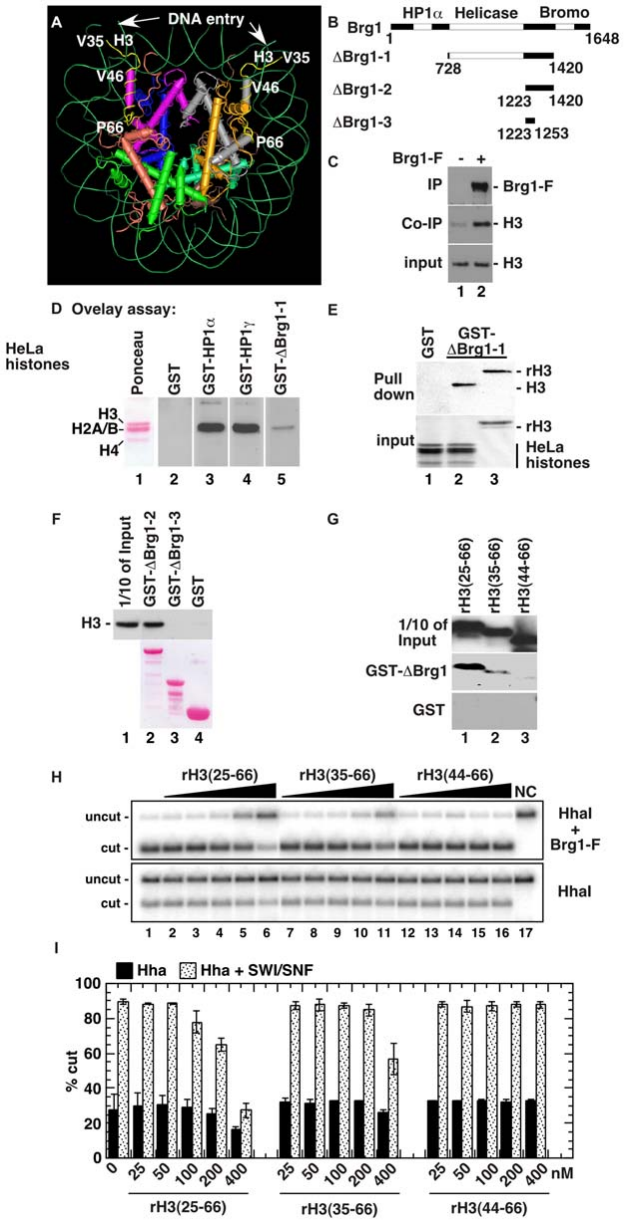
Brg1 binds histone H3. (A) Positioning on the nucleosome of the Shadock (V35 to P66, yellow) of histone H3 interacting with HP1 proteins. H3 histones are lilac and gray. (B) Schematic representation of the truncated Brg1 construct. HP1α: HP1α interaction domain [20]. Helicase: catalytic domain. Bromo: bromodomain. (C) Purified core histones were incubated in the absence (lane 1) or in the presence (lane 2) of recombinant flag-tagged full length Brg1. Immunoprecipitation was carried out with anti-flag antibodies. Immunoprecipitate was resolved by SDS-PAGE and analyzed by western blot using anti-Brg1 or anti-H3 antibodies. (D) Purified core histones were resolved by SDS-PAGE, blotted to nitrocellulose membrane and probed with indicated GST fusion-proteins. Bound proteins were detected with anti-GST antibodies and chemiluminescence. (E) Purified core histones or recombinant histone H3 (rH3) were incubated with GST or GST-ΔBrg1-1 bound to agarose beads. After washing, retained proteins were eluted, resolved by 4–12% gradient SDS-PAGE, and detected by western blotting with anti-histone H3 antibodies. (F) As in (E), with agarose beads bound to the indicated Brg1 truncation mutants. (G) As in (E), with the indicated H3 truncation mutants. (H) REA assays: 5S polynucleosome template at 1 nM was digested by *Hha*I in the presence or absence of hSWI/SNF pre-incubated with the indicated B10-tagged histone H3 polypeptides. Digestion products were separated on 1% agarose gels. NC: Not cut. (I) Quantification of three independent REA assays described in (H).

The Shadock region is located at the entry/exit site of the nucleosome but is partially hidden inside the nucleosomal barrel (Figure 2A). Interestingly, this region also includes the H3 αN helix previously shown to play an important role in nucleosome mobility [23] and mutations in this region were recently shown to affect chromatin remodeling by yeast SWI/SNF [24]. Besides, we found that histone H3 was co-immunoprecipitated with the hSWI/SNF catalytic subunit Brg1 in an *in vitro* assay (Figure 2C). We therefore investigated whether Brg1 would target this region during remodeling. In these experiments, we used a truncation mutant of the Brg1 protein centered on the ATPase domain (ΔBrg1-1, Figure 2B). This mutant, sufficiently short to be produced in *E. coli*, shows remodeling activity similar to full length Brg1 [25]. When expressed as a GST fusion, ΔBrg1-1 had affinity for histone H3 but not for other histones bound to nitrocellulose membrane (Figure 2D, lane 5). GST pull down assays further showed that ΔBrg1-1 bound to both purified and recombinant histone H3 (Figure 2E, lanes 2 and 3). Additional Brg1 deletion mutants showed that regions C-terminal of the helicase domain could mediate the interaction (Figure 2F). These regions were previously reported as essential for *in vitro* remodeling [25]. Binding properties of Brg1 to histone H3 could essentially be recapitulated with Brm, the alternative catalytic subunit of the hSWI/SNF complex (Figure S1E, S1F, S1G, S1H). H3 deletion mutants further revealed that the interaction of Brg1 with the histone was dependent on the region from aa 35 to 66 also involved in interaction with the HP1 proteins (Figure 2G, lane 2). Best binding was however achieved when this region was extended by 10 aa (aa 25 to 66, Figure 2G, lane 1).

To determine whether this interaction was important for chromatin remodeling, we used Restriction Enzyme Accessibility (REA) assays [26]. Nucleosomal arrays were assembled by the use of DNA templates consisting of two sets of five 5S nucleosome positioning sequences that flank DNA sufficient to assemble two nucleosomes, one of which overlaps a unique *Hha*I site. Accessibility of this site is increased in the presence of full length Brg1 and ATP, reflecting chromatin remodeling (in Figure 2H, lane 1 compare top and bottom panels). In these assays, we challenged the remodeling by Brg1 with H3 deletion mutants, reasoning that these polypeptides could interfere with the binding of Brg1 with its normal nucleosomal substrate. We observed a good correlation between the ability of the H3 mutants to inhibit the remodeling reaction and their ability to bind Brg1 (see the effect of 200nM and 400nM competing protein in Figure 2H and 2I, and compare with binding in Figure 2G). These observations show that the contact between Brg1 and H3 is important for remodeling.

### HP1α and HP1γ repress chromatin remodeling by hSWI/SNF *in vitro*

**Figure 3:**
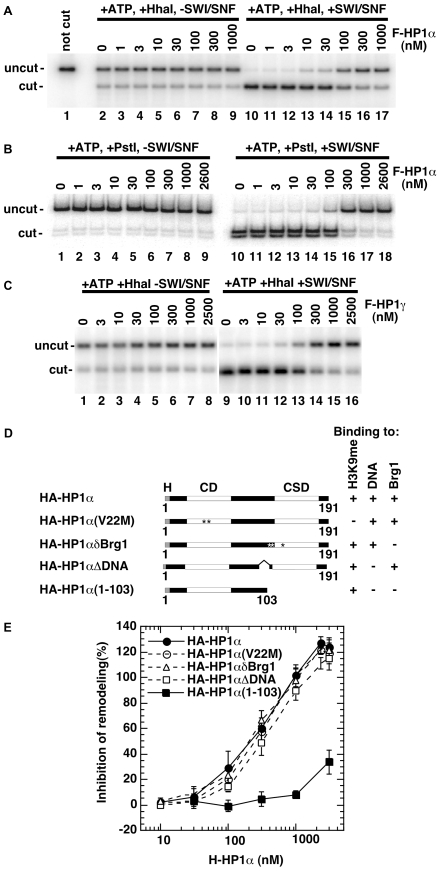
HP1α and HP1γ inhibits chromatin remodeling *in vitro*. (A) REA assay performed on a 5S polynucleosome template. Template at 1 nM was pre-incubated with indicated concentrations of recombinant F-HP1α (produced in baculovirus) before digestion by *Hha*I in the absence or presence of hSWI/SNF. At the end of the reaction (1hr), digestion products were separated on a 1% agarose gel. (B) REA assay performed as in (A) on a 202 bp mononucleosome template and the restriction enzyme PstI. Digestion products were separated on a 5% polyacrylamide gel. (C) REA assay performed as in (A) with indicated concentrations of recombinant F-HP1γ (produced in baculovirus). (D) Schematic representation of the HA-tagged HP1α point and deletion mutants produced in *E. coli*. Note that HA-HP1α(V22M) also carries a V21A mutation. (E) REA assays performed as in (A) with increasing concentrations of indicated HA-HP1α proteins. Values are averaged from 3 independent experiments.

The binding of both HP1 and Brg1/Brm proteins to the Shadock domain of histone H3 suggested a competition between these two sets of transcriptional regulators. To investigate this possibility, we tested the effect of HP1α on chromatin remodeling by the hSWI/SNF complex in REA assays. Addition of recombinant flag-tagged HP1α (F-HP1α) prevented the hSWI/SNF-dependent increase of *Hha*I accessibility without modifying the intrinsic accessibility of the restriction site (Figure 3A). Quantification showed that 50% inhibition of site accessibility was reached when nucleosomes, F-HP1α and hSWI/SNF were present at a molar ratio of approx. 1∶70∶10. In a similar assay F-HP1α also repressed remodeling by Brg1 and the truncated ΔBrg1 (Figure S2B).

We next analyzed the effect of F-HP1α on remodeling of a mononucleosome substrate. The mononucleosome was assembled on a 202 bp template containing a unique PstI site only 5% accessible in the absence of hSWI/SNF complex and ATP. As observed with the polynucleosomal template, F-HP1α inhibited remodeling of this substrate (Figure 3B). Repression was moderately less efficient as 50% inhibition was reached at a nucleosomes∶F-HP1α∶hSWI/SNF ratio of 1∶100∶10. Flag-tagged F-HP1γ also repressed hSWI/SNF remodeling with an efficiency similar to that of F-HP1α (Figure 3C). Measuring the kinetics of the repression revealed however that HP1γ was significantly slower than HP1α (Figure S2C).

We finally used the REA assay to test several HA-tagged HP1α constructs (Figure 3D). Consistent with a role for the CSD in the repression, truncation of the carboxyl terminal region abolished the repressing effect of HP1α on remodeling by hSWI/SNF. In contrast, mutants defective in either histone H3 K9me binding (HP1α V22M), DNA/RNA binding (ΔDNA), or interaction with Brm/Brg1 (HP1α δBrg1) were not affected in their ability to repress hSWI/SNF chromatin remodeling (Figure 3E). In addition, we observed HP1α-mediated repression on a nucleosomal array reconstituted with non-modified histones produced in *E. coli* (data not shown), confirming that binding of the CD to H3 K9me was not required for inhibition of hSWI/SNF activity.

### Antagonistic regulation of interferon-inducible genes by hSWI/SNF and HP1 proteins

**Figure 4:**
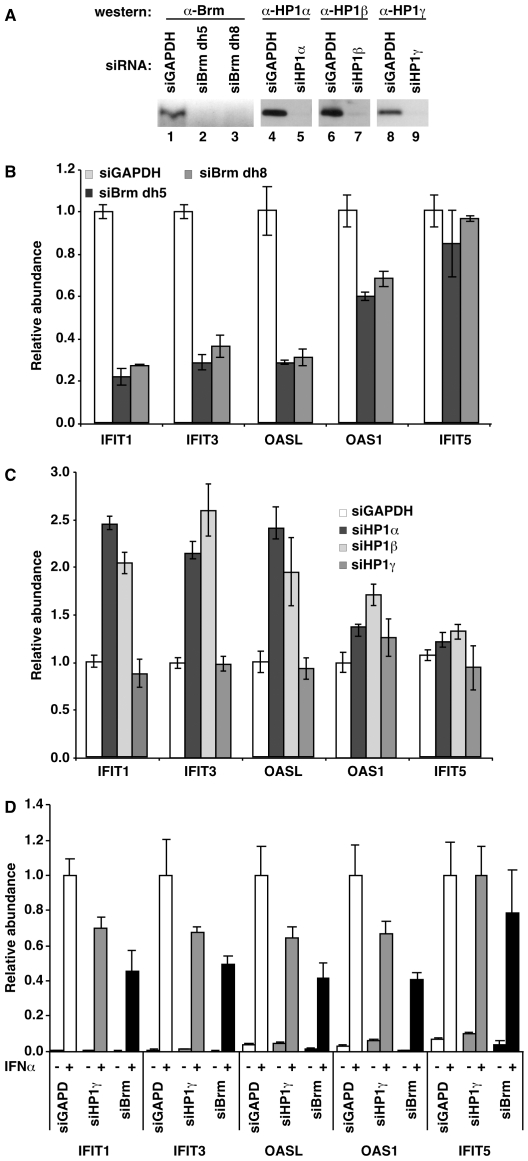
Brm and HP1α/HP1β have opposite effects on common target genes. (A) Western blots of extracts from HeLa cells transfected with the indicated siRNA and used for the preparation of the RNAs used in (B,C). The blots shown are representative of experimental triplicates. (B) HeLa cells were transfected with Brm siRNAs. The mRNA abundance from the indicated genes was measured by RT–qPCR and normalized to levels of HPRT. Values were averaged from experimental triplicates and normalized to levels of HPRT mRNA. (C) As in (B), with the indicated HP1 siRNAs. (D) As in (B), using HeLa HA-HP1γ cells (see Figure 5) and HP1γ or Brm siRNAs and a treatment with 0.5 nM interferon-α2 for either 0 or 10 hours as indicated.

We next set up to identify genes where the antagonism between Brg1/Brm and HP1 proteins could be visualized *in vivo*. Our attention was brought to interferon-regulated genes that are well-characterized hSWI/SNF targets and that, like the viral HIV1 LTR, are highly and rapidly inducible in response to outside stimuli [27]–[29]. To confirm the effect of hSWI/SNF on the transcription of these genes, we knocked down Brm in HeLa cells with two different siRNAs (Figure 4A, lanes 1–3). Brm was here preferred over Brg1 as Brm is degraded during each mitosis and is therefore very efficiently depleted with siRNAs [30]. This depletion resulted in repression of the interferon-inducible genes we tested, including IFIT1, IFIT3, OASL, and OAS1, with the exception of IFIT5 (Figure 4B). In contrast, these genes, again with the exception of IFIT5, were activated upon knock-down of either HP1α or HP1β (Figure 4A, lanes 4–7, and Figure 4C). These data show that several interferon-regulated genes rely on hSWI/SNF for their activation and on HP1α and HP1β for their repression.

Knock-down of HP1γ did not significantly affect the expression of the interferon-inducible genes in the absence of stimulation (Figure 4A, lanes 8–9 and 4C). However, we noted a moderate but reproducible decrease of the interferon-inducibility of the genes after depletion of HP1γ, again with the exception of IFIT5 (Figure 4D). These data are consistent with earlier reports showing an implication of HP1γ in efficient expression of some genes [5],[6].

Finally, we note that the activation of the interferon-inducible genes was observed only on 4 out of 5 tested genes, with HP1α and HP1β, but not with HP1γ or GAPDH siRNAs. In addition, our siRNAs were designed to minimize the interferon response [31]. We therefore ruled out a possible non-specific stimulation of the interferon pathway by the double-stranded siRNAs.

### Recruitment of HP1γ to chromatin is dependent on Brm/Brg1

Earlier studies have shown that HP1 proteins bind poorly to chromatin under physiological salt conditions while they associate tightly with destabilized nucleosomes from cells in S-phase [15],[17]. This would be consistent with nucleosomal structures preventing HP1 proteins to access either the DNA or the Shadock region inside the nucleosome barrel. It also suggests a possible effect of chromatin remodeling on the loading of HP1 proteins to chromatin.

**Figure 5:**
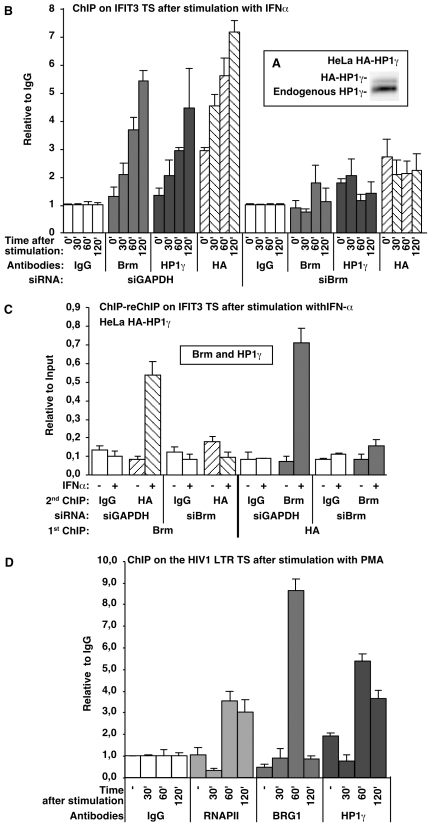
Brm facilitates recruitment of HP1γ to chromatin. (A) (Frame) Western blot with anti-HP1γ antibodies on total extract from HeLa expressing an HA-tagged version of HP1γ (HeLa HA-HP1γ). (B) Chromatin immunoprecipitation (ChIP): HeLa HA-HP1γ were stimulated with 0.5 nM interferon-α2 for the indicated times after siRNA-mediated knock down of either GAPDH or Brm. ChIP experiments were performed with the anti-Brm, anti-HP1γ, anti-HA epitope tag antibodies, or with total IgG as indicated. Enrichment in IFIT3 promoter chromatin was quantified by qPCR using primers spanning over the transcription start (TS) region. Values are averaged from 2 independent experiments. IgGs bring down approx. 1% of the input. (C) ChIP-reChIP: HeLa HA-HP1γ were stimulated with 0.5 nM interferon-α2 for 60′ after siRNA–mediated knock down of either GAPDH or Brm. Sequential ChIPs were carried out first with anti-Brm antibodies then with anti-HA antibodies or total IgG, or *vice versa*. Enrichment in IFIT3 promoter chromatin was quantified as in B. Values are averaged from 2 independent experiments. (D) ChIP: J-Lat A1 cells that carry a single integration of an HIV1–derived reporter construct were treated with phorbol ester PMA for the indicated times. ChIP experiments were performed with antibodies specific for RNAPII, Brg1, and HP1γ. Enrichment in HIV1 LTR chromatin was quantified by qPCR using primers spanning over the transcription start (TS) region. Values are averaged from 2 independent experiments.

To investigate whether hSWI/SNF activity could influence HP1 recruitment, we carried out chromatin immuno-precipitations (ChIP) on the IFIT3 promoter before and after knock down of Brm with siRNAs. To minimize the impact of the histone H3K9 methylation repression mark on recruitment of HP1 proteins, we followed the promoter during transcriptional activation and we concentrated our study on HP1γ that is not associated with repression of basal IFIT3 transcriptional activity (Figure 4C). The experiments were carried out with a HeLa-derived cell line stably expressing moderate levels of epitope-tagged HP1γ, thus allowing us to detect the protein with both anti-HP1γ and anti-HA tag antibodies (Figure 5A). Upon stimulation with interferon α, recruitment of both HP1γ and Brm increased (Figure 5B, siGAPDH). This is consistent with an implication of HP1γ in efficient expression of the IFIT3 gene as observed in Figure 4D. The recruitment of HP1γ was essentially abolished upon depletion of Brm (Figure 5B, siBrm). This decreased recruitment was not due to the silencing of the IFIT3 promoter, as Brm depletion reduces the transcriptional activity of the gene only approx. 2-fold (Figure 4D). ChIP-reChIP experiments further showed that Brm and HP1γ were present on the same chromatin fragments, suggesting that their recruitment is interdependent. This co-recruitment could be visualized with anti-Brm followed by anti-HA ChIP-reChIP as well as the inverse combination (Figure 5C).

We next investigated the timing of the recruitment of HP1γ and Brg1 on an integrated HIV1 LTR (Figure 5D). As mentioned in the introduction, transcriptional activation of this promoter results in eviction of HP1ß and increased recruitment of HP1γ [5]. ChIP analysis showed that recruitment of RNAPII, Brg1 and HP1γ peaks 60 min. after induction with PMA. Subsequently, low levels of Brg1 recruitment were restored while levels of RNAPII and HP1γ remained high. These observations are therefore compatible with transient recruitment of Brg1 helping the loading of HP1γ onto sites internal to the nucleosome. They also suggest that recruitment of HP1γ may limit the duration of hSWI/SNF-mediated chromatin remodeling during transcriptional activation.

### Brg1-mediated remodeling facilitates binding of HP1 proteins to nucleosomes

**Figure 6:**
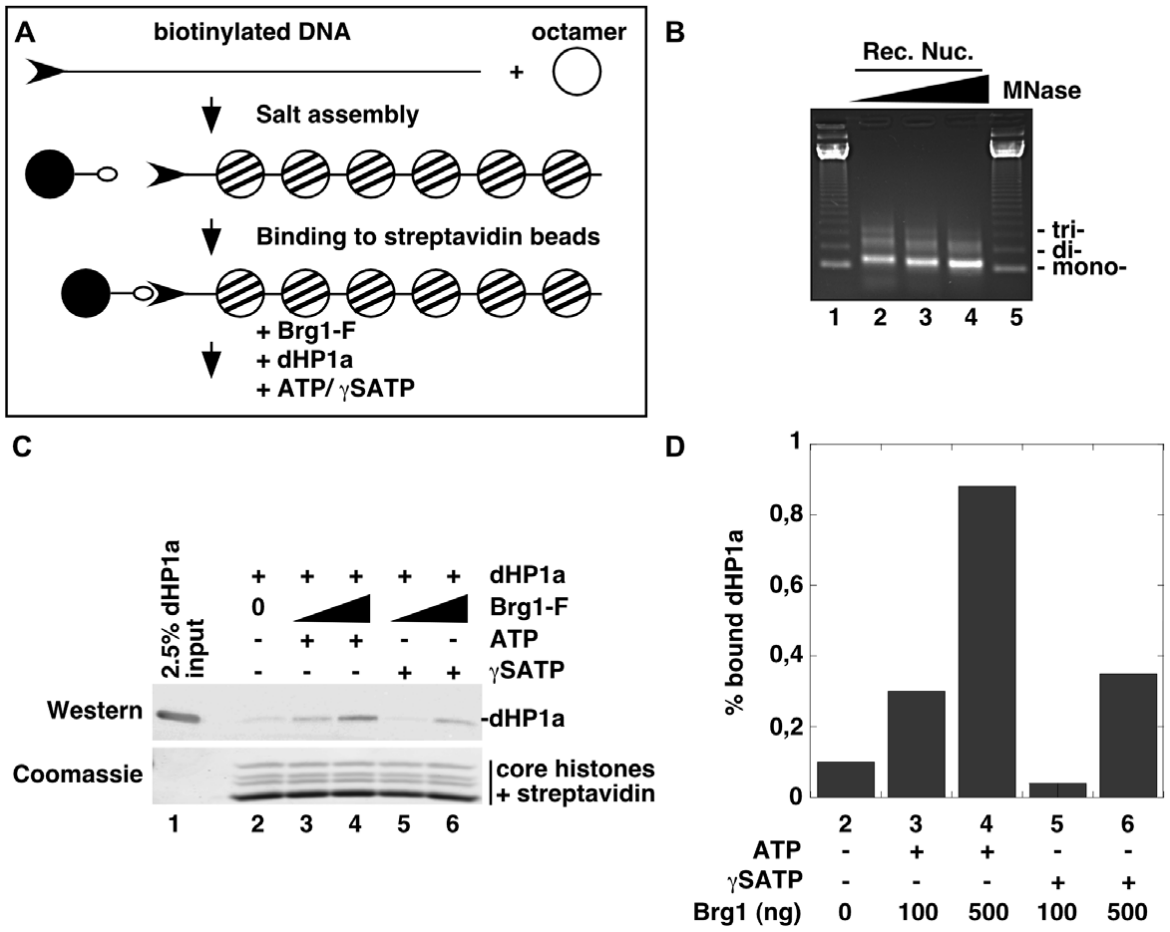
Brg1 remodeling facilitates binding of HP1 to nucleosomes. (A) Schematic representation of the chromatin reconstitution protocol. The DNA used for chromatin reconstitution is a linearized biotinylated fragment containing 12 repeats of the 5S nucleosome positioning sequence. (B) Micrococcal digestion pattern of salt-reconstituted chromatin with unmodified histones. (C) dHP1a was assayed for binding to chromatin in the absence or in the presence of Brg1 and either ATP or γSATP as indicated. (D) Quantification of experiment in (C).

To investigate *in vitro* whether chromatin opening could facilitate binding of HP1 proteins to nucleosomes, we finally used a recombinant nucleosomal array associated with streptavidin beads (Figure 6A and 6B). Consistent with earlier studies, this array assembled at relatively low ionic strength was poorly bound by *Drosophila* dHP1a (Figure 6C, lane 2 and [16],[32]). The *Drosophila* protein was here preferred because it could be more efficiently purified than its human counterparts and showed limited direct binding to Brg1 (Figure S3). The binding of this protein was significantly increased in the presence of full length recombinant purified Brg1 and ATP (Figure 6C, lanes 3 and 4). The effect was inhibited in the presence of γS-ATP, showing that it was dependent on the remodeling activity of Brg1 (Figure 6C, lane 5). Some binding was also observed in the presence of high levels of Brg1 and non-hydrolysable γATP (Figure 6C, lane 6), possibly explained either by a contamination of our preparation of Brg1 with ATP or by the interaction of dHP1a with Brg1.

## Discussion

We show here that, in addition to the contact of the CD with methylated H3K9, HP1 proteins use the CSD to associate with histone H3 at the level of the entry/exit point of the nucleosome barrel. This region on histone H3 that we termed Shadock is also contacted by Brg1 and Brm, the catalytic subunits of the hSWI/SNF complex, and chromatin remodeling can favor loading of HP1 proteins onto chromatin. We provide also evidence suggesting that this loading ultimately decreases the efficiency of hSWI/SNF remodeling.

FRAP experiments have shown that the CSD domain is required for the binding of HP1 proteins to native chromatin *in vivo*, with actually a stronger contribution than the CD when observation are made in euchromatic regions [33]. In addition, deletion of the CSD abolishes HP1-mediated transcriptional repression in transfection assays [34]. In the same type of experiment, we show that a point mutation in the CSD disrupting the interaction with the H3 Shadock region (I126F) is sufficient to interfere with the repressing activity. Furthermore, our REA assays show that the CSD is required for inhibition of chromatin remodeling by hSWI/SNF and that this remodeling complex contacts the same region on H3 as the HP1 proteins. Taken together, these observations strongly suggest that HP1 chromatin binding and repression activities are largely mediated by the histone binding activity of the CSD.

ChIP and ChIP reChIP assays show that Brm is required for the loading of HP1γ to the IFIT3 promoter during transcriptional activation. We note that on this promoter, we have another example of the switch from HP1α/HP1ß to HP1γ during transcriptional activation, also observed on the HIV1 LTR and on the Survivin promoter [5],[6]. This switch could suggest that, *in vivo*, histone H3K9 methylation is the determining factor for the recruitment of HP1α and HP1ß, while recruitment of HP1γ would be more dependent on chromatin opening by hSWI/SNF or the RNAPII. The role of HP1 proteins on active promoters is still enigmatic. The transient recruitment of Brg1 to the HIV1 promoter in sync with HP1γ recruitment shown in Figure 5D suggests that HP1 proteins could be involved in limiting remodeling on activated promoters. However, HP1 proteins may also have a role further downstream in connection with mRNA maturation [35].

The presence of HP1α on the IFIT1 and IFIT3 promoters and thereby the direct regulation of these genes by the HP1 protein was established by ChIP experiments (data not shown). However, the ChIP approach did not allow us to estimate the impact of hSWI/SNF remodeling on the recruitment of HP1α, as depletion of Brm leads to repression of the IFIT1 and IFIT3 promoters. Under these conditions, it was not possible to part between enhanced recruitment due to increased histone H3K9 methylation and decreased recruitment associated with reduced hSWI/SNF activity. However, our *in vitro* nucleosome-array binding assay suggests that HP1 proteins other than HP1γ can benefit from chromatin remodeling to bind nucleosomes. It must here be noted that this assay does not allow us to discriminate between histone and DNA binding. Nevertheless, repression of hSWI/SNF remodeling does not appear to rely on DNA binding as an HP1α mutant no longer binding DNA still represses. In addition, HP1γ does not bind to DNA in our hands and prefers nucleosomes (data not shown). Yet, it is efficient in repressing hSWI/SNF remodeling. Finally, we note that methylation of histone H3 on K9 is not required for repression of hSWI/SNF remodeling *in vitro*, further suggesting that the contact of the CSD with the Shadock, and not other contacts, is important for the repressing activity of HP1 proteins.

The interaction of HP1α CSD with histone H3 required HP1 dimerization and was disrupted by the mutation of V46 in the PXVXL-like sequence in the Shadock. It is therefore possible that this interaction can be structurally compared to that of other molecular partners of CSDs such as CAF1 or TIF1 proteins [36]. Interestingly, the rH3(35–66)V46A and rH3(44–66) mutants bind HP1γ but not HP1α, while H3 constructs with a full Shadock region bind both HP1 proteins. These observations show that HP1α and HP1γ have neighboring but distinct sites of interaction on the histone. While this manuscript was in revision, it was shown that phosphorylation of histone H3 on tyrosine 41 by JAK2 compromises the binding of the chromoshadow-domain of HP1α to an H3 peptide spanning from aa 31 to 56 [37]. This modification is outside the HP1γ binding site and it therefore seems possible that HP1α and HP1γ are differentially regulated by post-translational modifications in the H3 Shadock region.

The region of histone H3 contacted by the HP1 proteins also associates with the hSWI/SNF subunits Brg1 and Brm. This is compatible with earlier studies in yeast showing that residues K56 and L61 are involved in SWI/SNF recruitment [38],[39]. In yeast, it was suggested that the H3 αN helix is targeted and remodeled by the SWI/SNF complex [40]. More recently, mutagenesis within this region was shown to affect the efficiency of yeast SWI/SNF remodeling *in vitro* [24]. Our competition experiments with polypeptides mimicking the H3 αN helix further suggest that interaction of Brg1 with this region is essential for the chromatin remodeling activity of the hSWI/SNF complex. Consistent with this, the sequences downstream of the helicase domain of Brg1/Brm that mediate the interaction with the H3 Shadock domain have earlier been reported as essential for efficient remodeling *in vitro* [25]. We therefore speculate that the competing binding of Brg1/Brm and HP1 proteins to a same region of histone H3 during unwanted remodeling can at least in part explain the inhibiting effect of HP1 on chromatin opening by hSWI/SNF.

*In vitro* binding assays suggest that Brg1 has an affinity for the globular domain of H3 that is higher than that of HP1α (Figure S4). In a mechanism based on competition of Brg1 and HP1 proteins for binding to overlapping sites on histone H3, this difference in affinity may explain that a 7-fold excess of HP1α over Brg1 is required to obtain 50% inhibition of hSWI/SNF remodeling in the REA assays. It must finally be noted that the activity of the hSWI/SNF complex is also inhibited by the Polycomb Group (PcG) class II complex *in vitro* [26],[41]. This complex, involved in gene silencing, includes a CD protein that, like HP1 proteins, binds methylated histone H3 tails with a preference for methylation on K27 rather than K9 [42],[43]. However, Polycomb proteins contain no CSDs and it seems that PRC1 relies essentially on a structuring effect on the nucleosomal template, repressing remodeling by creating more condensed chromatin [44].

**Figure 7:**
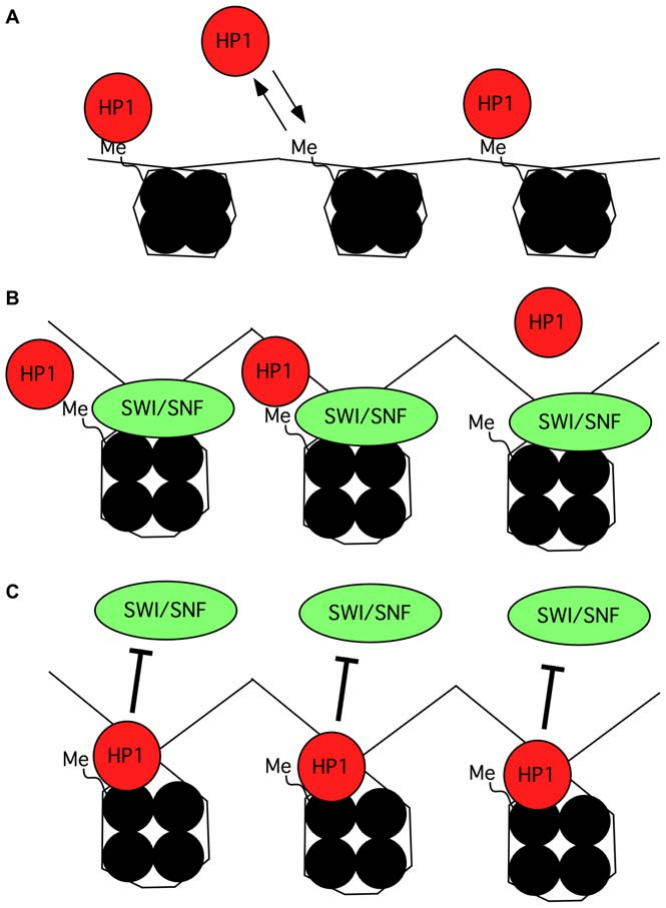
Model suggesting how HP1 proteins may gain access to internal nucleosomal regions and control remodeling by SWI/SNF. (A) When the chromatin is methylated on H3K9, HP1 is brought in the vicinity, attracted by the methylation mark. The interaction with the nucleosome is very dynamic. (B) Remodeling creates access to hidden HP1-binding sites on the globular domain of histone H3. (C) The exposure of the internal binding sites allows HP1 to detect the remodeling and to regulate it, gaining at the same time a more stable interaction with the nucleosome.

hSWI/SNF and HP1 proteins have many common target promoters including E2F1, human thymidine kinase, c-Myc, Sox2, Cyclin E, and the MMTV and HIV1 LTRs [5], [7], [45]–[51]. Likewise, we show here that several interferon-inducible genes that require hSWI/SNF for their activation are under the negative control of HP1α and HP1β. We note however that not all hSWI/SNF target genes we tested were affected by knock-down of HP1 proteins, including for example DraL and SPARC. These genes were both expressed at relatively high levels in the cells we used and may therefore not have any repressive structure on their promoter (data not shown). All considered, we suggest that on promoters where SWI/SNF functions as a repressor, the opening of the chromatin may, as previously suggested, be the event initiating HP1 stable recruitment [8],[20]. Where SWI/SNF functions as an activator, HP1 proteins recruited by methylated histone H3 tails may instead function as sensors of unwanted SWI/SNF activity, binding nucleosome domains uncovered by the remodeling, and thereby block the reaction (see proposed model in Figure 7). Finally, after the activation, additional HP1 proteins may be involved in controlling excessive remodeling activity. In that sense, HP1 proteins could very generally function as gatekeepers using the exposure of domains internal to the nucleosome to detect and restrict chromatin opening.

## Materials and Methods

### Transient transfection assays

MCF7 cells were transfected by calcium phosphate precipitation as previously described [52]. When indicated, 10^−6^ M dexamethasone (dex) was added to the medium. 40 hours post-transfection, luciferase assays were performed using the Promega luciferase kit, following the instructions of the manufacturer.

### Restriction enzyme accessibility (REA) assays

Flag-epitope tagged human HP1α and Brg1 were expressed in SF9 cells using a bac-to-bac expression system (Gibco) and purified on M2 anti-flag beads (Sigma). hSWI/SNF was purified from a Flag-tagged Ini1 HeLa cell line as described previously [53]. HA-tagged HP1α constructs were expressed in *E.coli* and purified using their additional 6xHis affinity tag. HA-HP1αΔDNA contains a deletion from aa 87 to 111. HA-HP1αΔBrg1 was previously described [20]. The polynucleosome template was assembled by gradient salt dialysis with HeLa core histones and a 5S arrays fragment [53],[54]. The mononucleosome template was assembled on a 202 bp TPT containing DNA fragment by salt dialysis [55] and incubated at 37°C for 3 hours before use. REA assays were performed as previously described [26].

### *In vitro* interaction experiments

GST-HP1 fusions and GST-ΔBrg1 were constructed in pGEX3X and pET41 plasmids respectively. HA-tagged HP1 and B10-tagged histone H3 were constructed in pET-28 and pET15b plasmids respectively that also providing a 6xHis affinity tag used for purification. Pull-down experiments were performed in ELB buffer (50mM HEPES pH 7, 250mM NaCl, 1mM EDTA, 0.1% NP40, 1xComplete protease inhibitor cocktail from Roche). Bound proteins were eluted in 100mM Tris pH 8, 20mM glutathione, resolved by SDS-PAGE and detected by western blotting using the B10 monoclonal mouse anti-estrogen receptor α antibodies (Euromedex ERB10-As) or anti-histone H3 rabbit polyclonal (Abcam ab1791). Overlay assays and nucleosome array binding assays were previously described [14],[16].

### RNA interference

siRNAs were synthesized by Dharmacon (ON-TARGET plus) : GAPDH (D-001830-01), Brm dh5 (J-017253-05), Brm dh8 (J-017253-08), HP1α (5′CACAAAUUGUGAUAGCAUU3′), HP1β (5′AGCUCAUGUUCCUGAUGAA3′) and HP1γ (5′AUCUGACAGUGAAUCUGAU 3′). siRNA were transfected into HeLa cells at 50 nM final concentration using DhamaFECT#1. Cells were harvested 3 days after transfection. RNAs were extracted using the Nucleobond RNA extract kit (Macherey-Nagel). mRNAs levels were quantified by real-time PCR after reverse-transcription performed at 50°C with SuperScriptIII (Invitrogene). Proteins were extracted as described previously [30] and detected by western blotting using anti-Brm (ab15597 Abcam) and anti-HP1α (2G9), anti-HP1β (1A9), and anti-HP1γ (1G6) from Euromedex.

### Chromatin immunoprecipitation (ChIP)

ChIP and ChIP-reChIP experiments were performed as previously described [56] using Jurkat J-Lat TAT-IRES-GFP clone A1 (NIH AIDS Research & Reference Reagent Program), or HeLa derived clones expressing HA-tagged HP1γ [5] and the following antibodies: anti-RNAPII (ab5095 Abcam), anti-Brg1 (2E12 Euromedex), anti-HP1γ (42S2 Millipore), anti-HA (12CA5), and anti-Brm (ab15597 Abcam). The eluted DNAs were detected by real-time PCR.

### Real-time PCR

Real-time PCR were performed with the SYBR Green kit Brilliant II (Agilent) reagents in a Mx3000 qPCR machine (Stratagene). The primers were the following:

IFIT1-F: ACACCTGAAAGGCCAGAATGAGGA,

IFIT1-R: TGCCAGTCTGCCCATGTGGTAATA,

IFIT3-F: AGCAAGAACATGCTGACCAAGCAG,

IFIT3-R: ACTTCAGTTGTGTCCACCCTTCCT,

OASL-F: ATGTTGGACGAAGGCTTCACCACT,

OASL-R: ATCTGTACCCTTCTGCCACGTTGA,

OAS1-F: GTTCTCCACCTGCTTCACAGAACT,

OAS1-R: CGAAATCCCTGGGCTGTGTTGAAA,

IFIT5-F: ATGGCCGCTTTCAGGAATTTCACC,

IFIT5-R: AGCACTTGTCAGTTTGGTGCGAAG,

HPRT-F: TATGGACAGGACTGAACGTCTTGC,

HPRT-R: TGAGCACACAGAGGGCTACAAT.

IFIT3_TS_F: AAAGCACAGACCTAACAGCACCCT,

IFIT3_TS_R: CATGATGGCTGTTTCCCTGCAGTT.

HIV1 TS primers were previously described [5].

## Supporting Information

Figure S1 Brm interacts with HP1α and histone H3, but not with HP1γ. (A) Schematic of Brm and the derived deletion mutants. The grey box symbolizes the GST purification tag. The ΔBrg1 construct is also indicated for comparison. (B) Brm co-immunoprecipitates with HP1α from HeLa total extract. PI: pre-immune serum. (C) The N-terminal region of Brm interacts with HP1α in a pull-down (lane 2), as previously shown for Brg1 (lane 3). (D) In a pull-down assay, the N-terminal region of Brm interacts with HP1α, but not HP1γ. (E) Overlay assay on HeLa nuclear extracts with the indicated proteins. (F) Overlay assays on recombinant (lane 1), recombinant truncated (lane 2), and purified histone H3 (lane 3), and on histone H4 (lane 4). The star indicates an impurity in the preparation of rH3(1–66). The ponceau correspond to the experiment shown in the top panel. (G) Overlay assay on purified bovine histones (purchased from Sigma) with the indicated Brm truncation mutants. In lanes 6 and 7, binding of Brm Cter is challenged with a 5-fold molar excess of either GST or GST-HP1α as indicated. (H) GST pull down assay with the indicated GST fusion proteins and purified bovine histones. Western with anti-histone H3.
(1.51 MB TIF)

Figure S2 HP1γ inhibits SWI/SNF remodeling with a reduced kinetic compared to HP1α. (A) Schematic representation of the truncated Brg1 construct. HP1α: HP1α interaction domain (Nielsen et al. 2002). Helicase: catalytic domain. AT+Br: AT hook and bromodomain. (B) 5S polynucleosome template at 1 nM was pre-incubated with indicated concentrations of recombinant Flag-tagged HP1α (produced in baculovirus) before digestion by *Hha*I in the absence or presence of either Brg1-F or ΔBrg1-F as indicated. Aliquots were removed at various times, quenched, de-proteinized and analyzed on 1% agarose gel. Rate constants were determined by fitting the entire reaction (fraction of uncut substrate versus time) to first-order (exponential decay) fits. (C) 5S polynucleosome template at 1 nM was pre-incubated with indicated concentrations of recombinant Flag-tagged HP1α or HP1γ (produced in baculovirus) before digestion by *Hha*I in the presence of hSWI/SNF. Aliquots were removed at various times, quenched, de-proteinized and analyzed on 1% agarose gel. Amounts of cut DNA was quantified by PhosphorImager. Data shown is compiled from two independent experiments.
(0.09 MB PDF)

Figure S3 Compared affinity of Drosophila dHP1a and human HP1α for Brg1. Purified Brg1-flag produced with baculovirus was incubated with agarose beads covered by either GST, GST-HP1α, or GST-dHP1a proteins as indicated. After washing, bound proteins were eluted, resolved on 4%–12.5% SDS-PAGE gradient gel and blotted on a nitrocellulose membrane. The membrane was stained with Ponceau (bottom panel) then incubated with anti-Brg1 2E12 monoclonal antibody (top panel). The figure shows that *Drosophila* dHP1a can bind human Brg1 but with a reduced affinity compared to human HP1α.
(0.26 MB PDF)

Figure S4 Compared affinity of Brg1 and HP1α for the globular domain of H3. Purified wt or mutant B10-tagged fragment of histone H3 (aa 35 to 66) was incubated with agarose beads covered by either GST-HP1α or GST-ΔBrg1 proteins as indicated. After washing, bound proteins were eluted, resolved on 12.5% SDS-PAGE and blotted on a nitrocellulose membrane. The membrane was stained with Ponceau (top panel) then incubated with anti-B10 monoclonal antibodies (bottom panel). The figure shows that approx. 50-fold excess of HP1α over Brg1 is required to obtain a similar binding to histone H3 in the region from aa 35 to 66.
(0.10 MB PDF)
