# Supplementary figures and images for: Interaction of HP1 and Brg1/Brm with the Globular Domain of Histone H3 Is Required for HP1-Mediated Repression
Source: PLoS Genet. 2009 Dec 11;5(12):e1000769. doi: 10.1371/journal.pgen.1000769 (PMC2782133; doi:10.1371/journal.pgen.1000769)

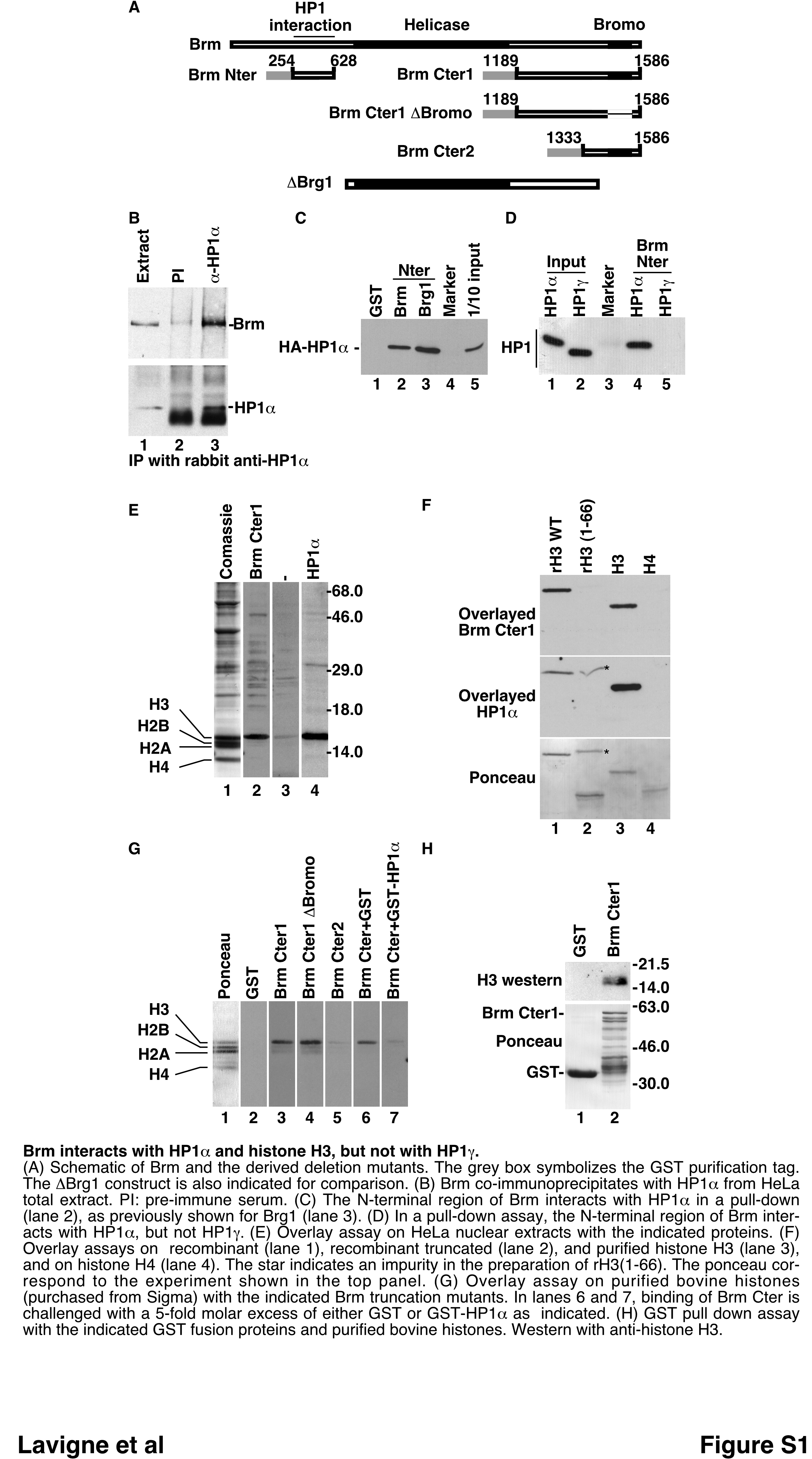

Supplement: Figure S1 — Brm interacts with HP1α and histone H3, but not with HP1γ. (A) Schematic of Brm and the derived deletion mutants. The grey box symbolizes the GST purification tag. The ΔBrg1 construct is also indicated for comparison. (B) Brm co-immunoprecipitates with HP1α from HeLa total extract. PI: pre-immune serum. (C) The N-terminal region of Brm interacts with HP1α in a pull-down (lane 2), as previously shown for Brg1 (lane 3). (D) In a pull-down assay, the N-terminal region of Brm interacts with HP1α, but not HP1γ. (E) Overlay assay on HeLa nuclear extracts with the indicated proteins. (F) Overlay assays on recombinant (lane 1), recombinant truncated (lane 2), and purified histone H3 (lane 3), and on histone H4 (lane 4). The star indicates an impurity in the preparation of rH3(1–66). The ponceau correspond to the experiment shown in the top panel. (G) Overlay assay on purified bovine histones (purchased from Sigma) with the indicated Brm truncation mutants. In lanes 6 and 7, binding of Brm Cter is challenged with a 5-fold molar excess of either GST or GST-HP1α as indicated. (H) GST pull down assay with the indicated GST fusion proteins and purified bovine histones. Western with anti-histone H3. (1.51 MB TIF) [file pgen.1000769.s001.tif]
