# Supplementary material for: Interaction of HP1 and Brg1/Brm with the Globular Domain of Histone H3 Is Required for HP1-Mediated Repression
Source: PLoS Genet. 2009 Dec 11;5(12):e1000769. doi: 10.1371/journal.pgen.1000769 (PMC2782133; doi:10.1371/journal.pgen.1000769)
Supplement: Figure S2 — HP1γ inhibits SWI/SNF remodeling with a reduced kinetic compared to HP1α. (A) Schematic representation of the truncated Brg1 construct. HP1α: HP1α interaction domain (Nielsen et al. 2002). Helicase: catalytic domain. AT+Br: AT hook and bromodomain. (B) 5S polynucleosome template at 1 nM was pre-incubated with indicated concentrations of recombinant Flag-tagged HP1α (produced in baculovirus) before digestion by HhaI in the absence or presence of either Brg1-F or ΔBrg1-F as indicated. Aliquots were removed at various times, quenched, de-proteinized and analyzed on 1% agarose gel. Rate constants were determined by fitting the entire reaction (fraction of uncut substrate versus time) to first-order (exponential decay) fits. (C) 5S polynucleosome template at 1 nM was pre-incubated with indicated concentrations of recombinant Flag-tagged HP1α or HP1γ (produced in baculovirus) before digestion by HhaI in the presence of hSWI/SNF. Aliquots were removed at various times, quenched, de-proteinized and analyzed on 1% agarose gel. Amounts of cut DNA was quantified by PhosphorImager. Data shown is compiled from two independent experiments. (0.09 MB PDF) [file pgen.1000769.s002.pdf]

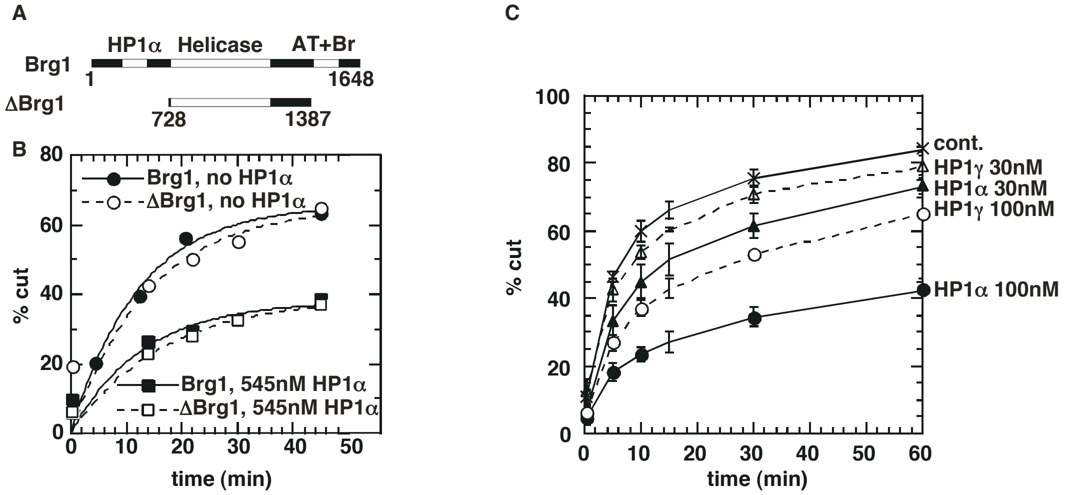

HP1γ inhibits SWI/SNF remodeling with a reduced kinetic compared to HP1α

A: Schematic representation of the truncated Brg1 construct. HP1α: HP1α interaction domain (Nielsen et al. 2002). Helicase: catalytic domain. AT+Br: AT hook and bromodomain.

B: 5S polynucleosome template at 1 nM was pre-incubated with indicated concentrations of recombinant Flag-tagged HP1α (produced in baculovirus) before digestion by *Hha*I in the absence or presence of either Brg1-F or ΔBrg1-F as indicated. Aliquots were removed at various times, quenched, de-proteinized and analyzed on 1% agarose gel. Rate constants were determined by fitting the entire reaction (fraction of uncut substrate vs. time) to first-order (exponential decay) fits.

C: 5S polynucleosome template at 1 nM was pre-incubated with indicated concentrations of recombinant Flag-tagged HP1α or HP1γ (produced in baculovirus) before digestion by *Hha*I in the presence of hSWI/SNF. Aliquots were removed at various times, quenched, de-proteinized and analyzed on 1% agarose gel. Amounts of cut DNA was quantified by PhosphorImager. Data shown is compiled from two independent experiments.

Lavigne et al

Figure S2
