# Supplementary material for: Interaction of HP1 and Brg1/Brm with the Globular Domain of Histone H3 Is Required for HP1-Mediated Repression
Source: PLoS Genet. 2009 Dec 11;5(12):e1000769. doi: 10.1371/journal.pgen.1000769 (PMC2782133; doi:10.1371/journal.pgen.1000769)
Supplement: Figure S3 — Compared affinity of Drosophila dHP1a and human HP1α for Brg1. Purified Brg1-flag produced with baculovirus was incubated with agarose beads covered by either GST, GST-HP1α, or GST-dHP1a proteins as indicated. After washing, bound proteins were eluted, resolved on 4%–12.5% SDS-PAGE gradient gel and blotted on a nitrocellulose membrane. The membrane was stained with Ponceau (bottom panel) then incubated with anti-Brg1 2E12 monoclonal antibody (top panel). The figure shows that Drosophila dHP1a can bind human Brg1 but with a reduced affinity compared to human HP1α. (0.26 MB PDF) [file pgen.1000769.s003.pdf]

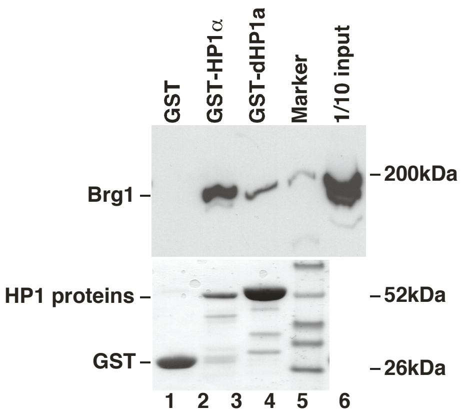

Compared affinity of *Drosophila* dHP1a and human HP1α for Brg1

Purified Brg1-flag produced with baculovirus was incubated with agarose beads covered by either GST, GST-HP1α or GST-dHP1a proteins as indicated. After washing, bound proteins were eluted, resolved on 4%-12.5% SDS-PAGE gradient gel and blotted on a nitrocellulose membrane. The membrane was stained with Ponceau (bottom panel) then incubated with anti-Brg1 2E12 monoclonal antibody (top panel). The figure shows that *Drosophila* dHP1a can bind human Brg1 but with a reduced affinity compared to human HP1α.
