# Supplementary material for: Interaction of HP1 and Brg1/Brm with the Globular Domain of Histone H3 Is Required for HP1-Mediated Repression
Source: PLoS Genet. 2009 Dec 11;5(12):e1000769. doi: 10.1371/journal.pgen.1000769 (PMC2782133; doi:10.1371/journal.pgen.1000769)
Supplement: Figure S4 — Compared affinity of Brg1 and HP1α for the globular domain of H3. Purified wt or mutant B10-tagged fragment of histone H3 (aa 35 to 66) was incubated with agarose beads covered by either GST-HP1α or GST-ΔBrg1 proteins as indicated. After washing, bound proteins were eluted, resolved on 12.5% SDS-PAGE and blotted on a nitrocellulose membrane. The membrane was stained with Ponceau (top panel) then incubated with anti-B10 monoclonal antibodies (bottom panel). The figure shows that approx. 50-fold excess of HP1α over Brg1 is required to obtain a similar binding to histone H3 in the region from aa 35 to 66. (0.10 MB PDF) [file pgen.1000769.s004.pdf]

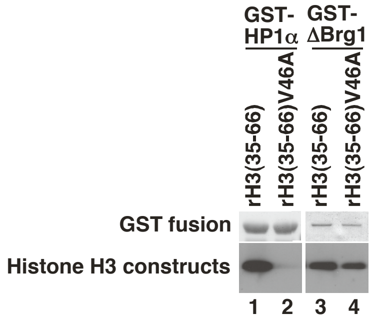

Compared affinity of Brg1 and HP1α for the globular domain of H3

Purified wt or mutant B10-tagged fragment of histone H3 (aa 35 to 66) was incubated with agarose beads covered by either GST-HP1α or GST-ΔBrg1 proteins as indicated. After washing, bound proteins were eluted, resolved on 12.5% SDS-PAGE and blotted on a nitrocellulose membrane. The membrane was stained with Ponceau (top panel) then incubated with anti-B10 monoclonal antibodies (bottom panel). The figure shows that approx. 50-fold excess of HP1α over Brg1 is required to obtain a similar binding to histone H3 in the region from aa 35 to 66.
